# 

**Published:** 1886-01

**Authors:** 


### CONTENTS.

### COMMUNICATIONS.

Association Work ........ 5
Address ........ 10
Geographical Distribution of Dental Caries ........ 14
Nervousness, Hereditary and Acquired ........ 19
Treatment of Third Molars ........ 24

### PROCEEDINGS.

Ohio State Dental Society ........ 26
Additional Thoughts on Pyorrhœa Alveolaris ........ 27
Pyorrhœa Alveolaris Infection ........ 29
The Eruption of the Permanent Teeth ........ 39

### CORRESPONDENCE.

Cocaine ........ 42
Salmagundi ........ 44

### EDITORIAL.

Herbst's Method ........ 47
International Medical Congress ........ 50
Midland Railway ........ 54
Obituary ........ 54

